# How to Measure Grana – Ultrastructural Features of Thylakoid Membranes of Plant Chloroplasts

**DOI:** 10.3389/fpls.2021.756009

**Published:** 2021-10-06

**Authors:** Radosław Mazur, Agnieszka Mostowska, Łucja Kowalewska

**Affiliations:** ^1^Department of Metabolic Regulation, Institute of Biochemistry, Faculty of Biology, University of Warsaw, Warsaw, Poland; ^2^Department of Plant Anatomy and Cytology, Institute of Plant Experimental Biology and Biotechnology, Faculty of Biology, University of Warsaw, Warsaw, Poland

**Keywords:** chloroplast, grana, granum stack, measurements, thylakoid membranes, transmission electron microscopy, ultrastructure

## Abstract

Granum is a basic structural unit of the thylakoid membrane network of plant chloroplasts. It is composed of multiple flattened membranes forming a stacked arrangement of a cylindrical shape. Grana membranes are composed of lipids and tightly packed pigment-protein complexes whose primary role is the catalysis of photosynthetic light reactions. These membranes are highly dynamic structures capable of adapting to changing environmental conditions by fine-tuning photochemical efficiency, manifested by the structural reorganization of grana stacks. Due to a nanometer length scale of the structural granum features, the application of high-resolution electron microscopic techniques is essential for a detailed analysis of the granum architecture. This mini-review overviews recent approaches to quantitative grana structure analyses from electron microscopy data, highlighting the basic manual measurements and semi-automated workflows. We outline and define structural parameters used by different authors, for instance, granum height and diameter, thylakoid thickness, end-membrane length, Stacking Repeat Distance, and Granum Lateral Irregularity. This article also presents insights into efficient and effective measurements of grana stacks visualized on 2D micrographs. The information on how to correctly interpret obtained data, taking into account the 3D nature of grana stacks projected onto 2D space of electron micrograph, is also given. Grana ultrastructural observations reveal key features of this intriguing membrane arrangement, broadening our knowledge of the thylakoid network’s remarkable plasticity.

## Grana as Basic Structural Units of the Chloroplast Thylakoid Network in Plants

Grana are essential structural features of the chloroplast thylakoid network, which are specific for plants. They are both confined structures characterized by a distinct molecular composition and, simultaneously, continuous elements of intertwined stroma-grana thylakoid network. Other photosynthetic organisms do not have a clear division between stacked grana and loosely arranged stroma thylakoid (ST) domains. Cyanobacteria possess unstacked photosynthetic membranes forming fascicular, radial, or parallel arrangements (Mareš et al., 2019). Similarly, red algae also do not exhibit thylakoid stacking, while brown algae and diatoms contain appressed membranes grouped by 2 or 3 (Bertrand, 2010). In green algae, the thylakoid membranes are organized into clearly differentiated stacked and unstacked regions but without highly structured multiple membrane layers characteristic for plant grana (Engel et al., 2015). Some authors describe thylakoid membrane stacks of late branching green algae taxa *Coleochaetales* and *Charales* as grana; however, based on the widely accepted evolutionary hypothesis, grana evolved after land colonization and therefore, are unique for plants (Gunning and Schwartz, 1999; Larkum and Vesk, 2003; Mullineaux, 2005). It is worth noticing that although thylakoid membranes of different plant groups, from Bryophytes to Angiosperms, show high variability of photosynthetic complexes supramolecular organization, their grana exhibit similar nano-morphology (Chen et al., 2018b).

**Figure 1:**
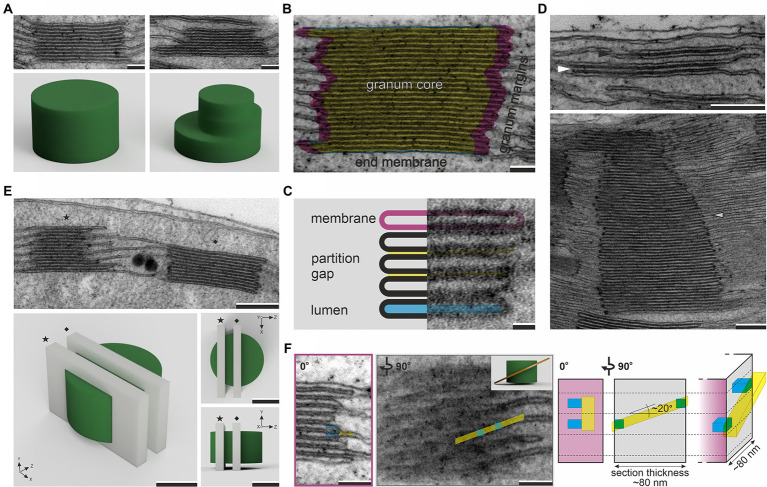
Electron micrographs of regular (left column) and irregular (right column) grana structures with corresponding 3D models showing the hypothetical spatial representation of visualized stacks (**A**). Three main ultrastructural components of granum stack – granum core (yellow), granum margins (pink), end membranes (blue; **B**). Basic compartments of stacked thylakoids – membrane (pink), partition gap (yellow), and lumen (blue; **C**). Electron micrographs showing two-layer thylakoid membrane stack, i.e., “membrane overlap” (black arrowhead), the lowest possible granum built by three thylakoid layers (white arrowhead), and extremely high granum stack composed of over 70 thylakoid layers (gray arrowhead; **D**). Electron micrograph and corresponding 3D models showing two different *z*-axis planes (star – tangential cut, diamond – central cut) of random granum sectioning; note that section planes (light gray cuboids) visible on 3D granum renders (green) represent 70-nm-thick ultrathin sections (**E**). Electron micrographs showing connection of neighboring grana thylakoids through stroma thylakoid (yellow) in the granum marginal region (blue) as visualized from two different angles (inset in the right corner of the second image presents a simplified 3D model of helical grana-stroma thylakoid arrangement); right side of the panel shows a scheme of the connection region visible in the perspective view and two orientations corresponding to presented micrographs; for ultrathin section with thickness between 70 and 90nm and angle of stroma thylakoid staggering ~20°, connection of two neighboring grana thylakoid layers can be observed in one section and visible as a fork-like structure on the sample projection (transmission electron microscopy (TEM) image; **F**). Note that various angles of granum section showed in panels **(E)** and **(F)** are presented on different grana stacks; electron micrographs were obtained from fully developed chloroplasts of *Arabidopsis thaliana* (**A–F**, with the exception of the lower image in panel **D**) and *Ficus elastica* (lower image on panel **D**); scale bar=100nm **(A,B,F)**, 20nm **(C)**, 250nm **(D,E)**.

From the structural point of view, grana might be described as stacks of discoidal-shaped thylakoids; however, such a general definition is insufficient for detailed qualitative and quantitative analysis of grana structure. A single granum stack is usually composed of 5–25 thylakoid layers with diameters between 300 and 550nm. The model granum structure is built by thylakoid membranes with the same diameter forming a perfect cylindrical shape. However, in most plant species and specific environmental conditions, the grana structures are highly irregular – with a variable diameter of thylakoid layers and their shift in the lateral plane (Figure 1A). It is difficult to structurally distinguish the individual grana stacks with high confidence; therefore, a precise procedure formulation is required (see “Grana Ultrastructural Parameters”).

General granum definition also does not determine the boundary of the granum in the vertical plane. No limit of the maximal granum height exists; many plant species, especially shade-grown ones, can form grana composed of over 50 layers (Anderson et al., 1973; Chow et al., 2005). Nonetheless, the minimal number of stacked layers that can be considered granum is questionable. The granum stack is built of its core, margins, and end-membranes (Figure 1B). These components are characterized by the specific lipid–pigment–protein composition (reviewed in Ruban and Johnson, 2015; Koochak et al., 2019). The boundary of the granum in the lateral plane is set by highly curved membrane regions called grana margins, with peripheries called “curvature domains” that can be biochemically separated (Trotta et al., 2019). Some authors propose a more strict definition of grana margins based on biochemical studies, describing them as an interface between appressed and non-appressed regions only (Rantala et al., 2020). Thylakoids resemble flattened vesicles; they are composed of two membranes and an inner aqueous compartment called “lumen.” Neighboring thylakoids in the stack are partitioned by a thin layer of stroma compartment called “stromal gap,” “interthylakoid stromal space,” or “partition gap” (Figure 1C). In the case of core thylakoids, both membranes are identical, while grana end-thylakoids have a heterogeneous architecture; the inner membrane is similar to that of the core thylakoids and the outer one to the ST membrane (Anderson et al., 2012). Considering all this, the lowest granum stack, which contains all types of structural components, has to be built by at least three thylakoid layers. Already in early ultrastructural studies, the two-layer membrane stacks were described as “membrane overlaps” pointing to a different structural assignment of such arrangements (Gunning and Steer, 1975; Figure 1D).

An accurate understanding of the granum structure enables the determination of reliable measuring protocols necessary to provide comparable results obtained by different researchers. Detailed analysis of grana structure provides important information about the thylakoid membrane remodeling forced by plant ontogenesis (Kowalewska et al., 2016; Armarego-Marriott et al., 2019; Pipitone et al., 2021), light intensity and quality (Rozak et al., 2002; Yamamoto et al., 2014; Demmig-Adams et al., 2015; Schumann et al., 2017; Flannery et al., 2021), and other environmental factors (Fq et al., 2012; Jiang et al., 2017; Chen et al., 2018a; Zechmann, 2019; Mazur et al., 2020). However, structural analysis indirectly indicates the organization and efficiency of the photosynthetic light reaction machinery (Pribil et al., 2018; Mazur et al., 2019; Wood et al., 2019; Hepworth et al., 2021) and photonic effects in the thylakoid network (Capretti et al., 2019).

Structural grana analysis using mutants with an aberrant composition of thylakoids is particularly helpful in understanding the role of lipid–pigment–protein components in the self-organization of different membrane shapes, such as curved, flat, and stacked regions (Fristedt et al., 2009; Armbruster et al., 2013; Mazur et al., 2019; Bykowski et al., 2021; Gupta et al., 2021; Raven, 2021). A necessary condition, though not sufficient, to define a given chloroplast-localized structure as granum is membrane stacking. The balance between attractive van der Waals forces, repulsive electrostatic and hydrostructural forces was described as crucial for maintaining grana stacking. The abundance, stability, and surface charge of thylakoid membrane components mainly mediate such balance (Puthiyaveetil et al., 2017).

Several thylakoid membrane components have been recognized to play a crucial role in the fine-tuning of grana structure. Numerous studies showed that changes in the ratio of antenna light-harvesting complexes (LHCII) and core proteins of photosystem II (PSII) induce grana size remodeling. Depletion of chloroplast-encoded PSII subunits caused the formation of “super-grana” containing dozens of membranes (Belgio et al., 2015), while lack of Lhcb1 and Lhcb2 antenna proteins resulted in a significant decrease in grana height (Andersson et al., 2003; Anderson et al., 2012; Pietrzykowska et al., 2014; Nicol et al., 2019). Small grana size is also typical for the *chlorina* mutants, characterized by reduced chlorophyll *b* content (e.g., Kim et al., 2009). Tuning of grana structure was also linked with posttranslational modifications of photosynthetic proteins, e.g., increased polyamination of Lhcb, resulting from overexpression of plastidial transglutaminase, caused the formation of extremely high grana stacks (Ioannidis et al., 2009; Ioannidis et al., 2012). In contrast, decreased acetylation of photosynthetic proteins (Arabidopsis *nsi* mutant) induced the formation of lower stacks compared with wild-type plants (Koskela et al., 2018), and lack of PSII core protein phosphorylation (Arabidopsis *stn8*, *stn7stn8* mutants) resulted in increased grana diameter (Fristedt et al., 2009).

Moreover, a family of structural membrane proteins – CURVATURE THYLAKOID 1 (CURT1), were recognized to mediate the diameter of grana stacks in a dosage-dependent manner and facilitate membrane curvature at the grana margins (Armbruster et al., 2013; Pribil et al., 2018). The grana-localized REDUCED INDUCTION OF NON-PHOTOCHEMICAL QUENCHING (RIQ) proteins regulate the grana height and probably link the grana structure with the organization of LHCII (Yokoyama et al., 2016). All acyl lipid components of thylakoid membranes were proved to be important in maintaining proper grana sizes; however, only monogalactosyldiacylglycerol role in the formation of helical grana arrangements was shown (Yu and Benning, 2003; Mazur et al., 2019). We have also recently presented that increased lutein to carotene ratio causing membrane rigidification results in hampered grana membrane folding (Bykowski et al., 2021). In all of these studies, ultrastructural transmission electron microscopy (TEM) analysis was essential to understand structural role of particular membrane components in the grana self-organization process.

## Methods Used in the Visualization of Granum Morphology and Their Limitations

Efficient measurements of grana structural parameters require high-quality visualization of the thylakoid network. The catalog of suitable microscopy methods is limited due to the dimensions of the grana stacks. In general, these methods can be divided into two groups (i) enabling *in vivo* analysis but with lower resolution, and (ii) high-resolution methods requiring sample fixation.

*In vivo* methods are mainly based on the detection of chlorophyll autofluorescence (reviewed in Kowalewska et al., 2019). Their advantage lies in the precise tracking of membrane remodeling triggered by different factors. Still, they fail in detailed grana visualization at the level of particular thylakoid layers. Typical grana structural parameters obtained using an *in vivo* approach are granum diameter (Uwada et al., 2017; Wood et al., 2019; Hepworth et al., 2021), also defined as full-width at half-maximum fluorescence intensity of the fluorescent spots (grana; Herbstova et al., 2012; Iwai et al., 2018; Wood et al., 2018; Flannery et al., 2021), and parameters describing the whole network. These parameters include: the number of grana stacks per chloroplast (Wood et al., 2018, 2019; Mazur et al., 2019), their distribution (Herbstova et al., 2012; Chen et al., 2014), and average grana sizes determined indirectly by the surface/volume ratio for 3D models of chlorophyll fluorescence (Bykowski et al., 2021).

High-resolution TEM, although requiring sample fixation, remains the most favored method to study the grana morphology due to the nanometer length scale of the grana structural details. The thylakoid ultrastructure might also be assessed using small-angle scattering methods that enable a noninvasive analysis of high volume samples (Ünnep et al., 2017; Jakubauskas et al., 2019; Ünnep et al., 2020; Zsiros et al., 2020; Jakubauskas et al., 2021). Only periodic membrane attributes can be registered, while no information on grana diameter or details of membrane connections could be revealed. TEM, however, gives access to a broader range of grana structural parameters defined and described in “Grana Ultrastructural Parameters” of this mini-review.

Regardless of the numerous advantages of the TEM method in the grana structure studies, particular sample preparation conditions should be considered for a reliable analysis. Due to the relatively small area of TEM analysis, it is essential to control the region of sampling. Chloroplasts of the leaf mesophyll of mono- and dicotyledonous plants do not form a uniform group of organelles. Their thylakoid network is characterized by different structural parameters depending on the leaf age and cell position within the leaf blade (Avramova et al., 2015; Gugel and Soll, 2016). Similarly, a unified time of sample collection is also essential. Suppose sampling throughout the light-dark cycle is not required. In that case, the most favorable time for sample fixation is at the end of the dark phase when the starch grains are, in most cases, degraded, enabling proper observation of the thylakoid network. This approach applies both for samples fixed using chemical and cryo-protocols. High-pressure freezing combined with the freeze-substitution method is particularly susceptible to starch grains whose presence during the procedure leads to the local thylakoid swelling near starch deposits (McDonald, 2014; Armarego-Marriott et al., 2019).

## Interpretation of a 3D Grana Structure Projected onto 2D Space

Although electron microscopy techniques enable a volumetric analysis of samples (electron tomography, serial block-face scanning electron microscopy, or focus ion beam scanning electron microscopy), these techniques are time- and money-consuming. For instance, visualization of the 3D structure of granum using electron tomography requires a multistep procedure composed of data acquisition, alignment, reconstruction, segmentation, and visualization (reviewed in Daum and Kuhlbrandt, 2011; Otegui and Pennington, 2018; Staehelin and Paolillo, 2020). Such extensive workflow limits the possibility of obtaining large data sets and, therefore, reliable quantitative analysis of particular structural parameters. In contrast, 2D TEM analysis is more accessible for researchers and enables the creation of relatively large data sets. However, it is essential to acknowledge that 2D analysis of 3D objects with complicated spatial structures is not straightforward, and the random nature of sample cutting has to be taken into account. The analysis of granum diameter on 2D sections is prone to chord error. It cannot be established whether the observed section shows the diameter or any other chord of the discoidal granum (Figure 1E). Therefore, large values of standard deviation are typical for the granum diameter measurements, which points to the necessity of analyzing big data sets for reliable comparison between samples. Another common issue in proper analysis of 2D grana images is related to the interpretation of the structure of grana-stroma thylakoid connections. In these regions, some authors show “fork-like connections” of ST with two neighboring layers of grana stack (fret-like protrusions) using 2D TEM projections (Shimoni et al., 2005; Koochak et al., 2019). However, it should be stressed that such structures are most probably only local phenomena in the *z*-axis of the specimen, which, for 3D models, are parts of STs staggering between granum layers forming pseudo-helical arrangement in the nearest stack surrounding (Mustárdy et al., 2008; Austin and Staehelin, 2011; Kowalewska et al., 2016; Bussi et al., 2019). The typical ultrathin section is 70–90nm thick; if the granum-connected ST membrane shifts at an angle of around 20° (Austin and Staehelin, 2011; Bussi et al., 2019), membrane staggering between two neighboring layers might be observed within one specimen (for details see Figure 1F). TEM images are projections of the visualized sample; therefore, it is impossible to establish the membrane’s *z*-axis position inside the sample, and the risk of misinterpretation is significant.

## Grana Ultrastructural Parameters

The first measurements of grana ultrastructural parameters were performed, *in situ*, with the help of mechanical instruments – curvometers on printed electron micrographs of chloroplasts (similarly to measurements on maps). The length of grana and stroma thylakoids was measured per randomly chosen unit area (1μm^2^) of each chloroplast cross section. The number of thylakoids per granum was also determined (e.g., Brangeon, 1973; Mostowska, 1986). Such basic parameters are also frequently assessed in current microscopy studies using digital micrographs and image analysis software of choice, followed by different approaches in data presentation and appropriate statistical analyses (e.g., Rozak et al., 2002; Anderson et al., 2012; Pietrzykowska et al., 2014; Pribil et al., 2018; Wood et al., 2018; Nicol et al., 2019; Li et al., 2020).

**Figure 2:**
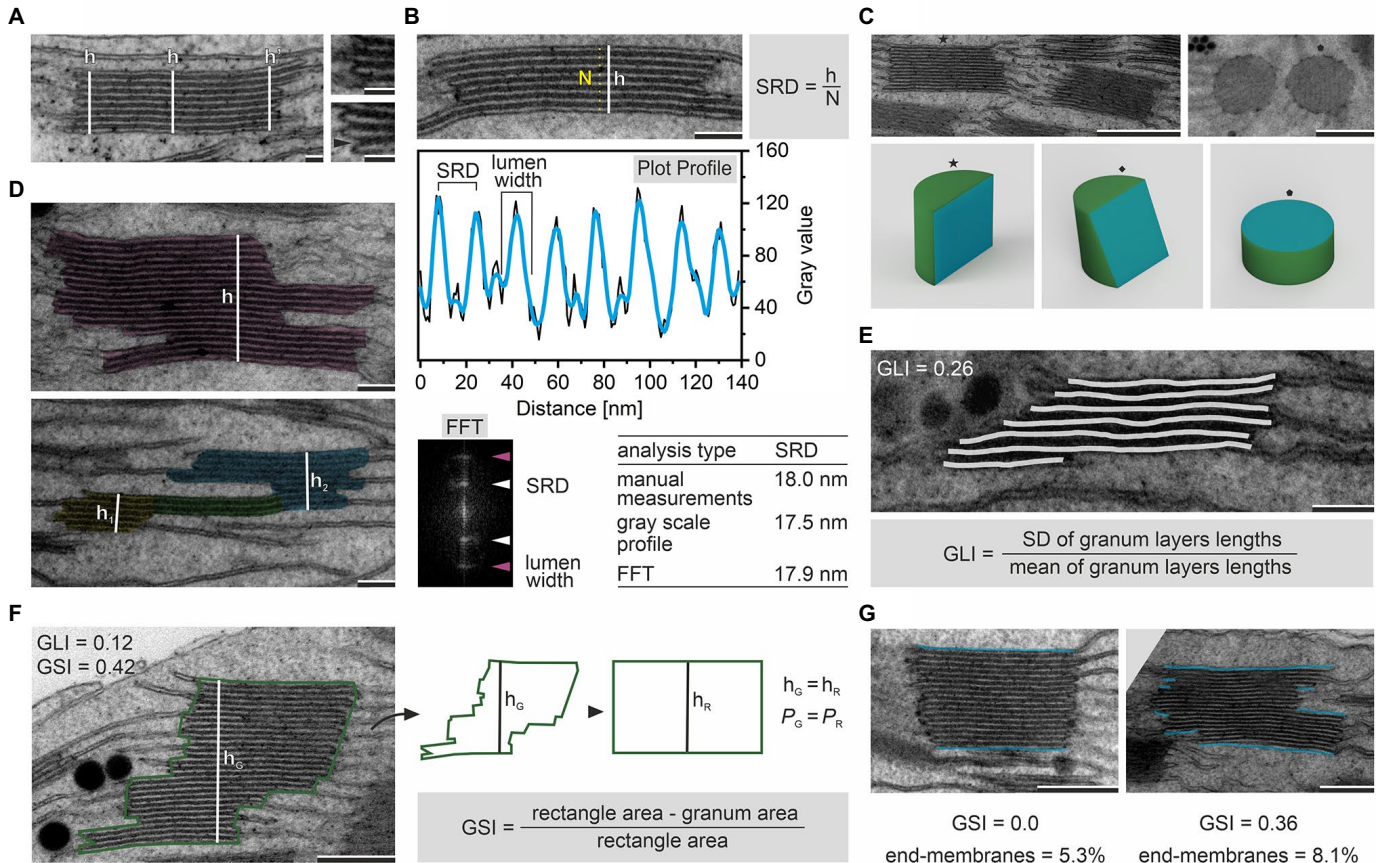
Determination of granum height (h) by measurements in central and marginal region of the stack; the upper inset shows typical marginal region, the lower inset presents membrane bending in the granum margin resulting in the local increase in stack height; note that such local increase might also be registered in the case of the lumen margin swelling **(A)**. Various image-analysis approaches used to calculate the Stacking Repeat Distance (SRD) parameter; the manual method requires measurement of the granum height (h), thylakoid layers counting (N), and application of the given formula; semiautomatic approach is based on the analysis of gray scale intensity profile obtained using, e.g., ImageJ Plot Profile function on the manually marked region (gray – raw data, blue – plot smoothed using Savitzky–Golay filter); Fast Fourier Transformation (FFT) analysis of granum periodicity enabling single-step automated SRD calculation; note that all calculated SRD values presented in the table were obtained by the analysis of the granum micrograph showed in the upper part of this panel **(B)**. Appearance of granum ultrastructure depends on the angle of granum section; cutting planes (blue) are presented on simplified rendered grana 3D models (star – parallel cut, diamond – shifted cut, pentagon – top cut); note that only sections parallel to the vertical granum axis enable reliable analyses of SRD parameter **(C)**. Exemplary images showing irregular membrane stack (pink) having one common height (h; upper micrograph) and membrane stack composed of two distinct sections (yellow, blue) of different heights (h_1_, h_2_) connected by a three-layer sector (green; lower micrograph); note that according to the definition used in this manuscript membrane stacks with one common height only are in the single granum category **(D)**. Marking of granum thylakoid lengths (light gray) on an exemplary granum micrograph; these values are necessary to calculate the Granum Lateral Irregularity (GLI) according to the provided formula; note that GLI value given in the upper left corner was calculated from the presented image **(E)**. Marking of the granum height (h_G_, white) and the perimeter (P_G_, green); these values are necessary to calculate the cross-sectional granum area and also area of the rectangle having identical height and perimeter as the measured granum; both area values are necessary to calculate the Granum Cross-Sectional Irregularity (GSI) according to the provided formula; note that GSI and GLI can be significantly different in one granum stack **(F)**. Images of two grana stacks with different GSI values and the end-membrane region marked (blue); higher value of GSI parameter corresponds to an increase in the ratio of the end-membrane length to the total membrane length within particular granum (expressed in %; **G**). Note that various angles of granum sections showed in panel **(C)** are presented on different grana stacks; electron micrographs were obtained from fully developed chloroplasts of *Arabidopsis thaliana* (left images on **A** and **C**; **B**,**D**,**E**; right image on **G**) and *Pisum sativum* (insets on **A**; right image on **C**; **F**; left image on **G**); scale bar=50nm **(A)**, 100nm (**B,D,E**), 250nm (**F,G**), 500nm (**C**). Ultrastructural features of grana were calculated with the help of ImageJ software (Abramoff et al., 2004).

Several structural parameters describe the vertical direction of the granum ultrastructure. The most basic one is the granum height. It is established by measuring the distance between the top and bottom layers of the granum end-membranes. Such distance has to be measured perpendicular to the granum lateral plane (Figure 2A). Significant variability in the granum height was registered in plants exposed to different light conditions and connected to the proportion between PSII core and antennae complexes (reviewed in Allen and Forsberg, 2001; Waters and Langdale, 2009; Anderson et al., 2012). The granum height might also be affected by modified physical properties of thylakoid membranes forced by changes in their pigment–protein composition, influencing membrane folding capabilities (Bykowski et al., 2021). Moreover, significant changes in the height of granum stacks were observed in the second phase of the chloroplast biogenesis in different plant species, together with the accumulation of associated photosynthetic proteins and capacity (Armarego-Marriott et al., 2019; Pipitone et al., 2021). In the most general approach, height in the middle of the granum diameter is obtained; however, if the thylakoid thickness is not constant throughout the diameter of the stack, additional measurements have to be taken (for details, see Figure 2A). Such analysis enabled, e.g., observation of swelling and/or bending of the grana marginal regions in plants exposed to high-light conditions to empower more efficient D1 protein turnover (Herbstova et al., 2012; Yoshioka-Nishimura et al., 2014; Kirchhoff, 2019).

Measurement of height combined with the counting of the thylakoid layers that build a particular granum stack enables calculation of the so-called Stacking Repeat Distance (SRD), also named “Repeat Distance,” which represents the average thickness of the thylakoid with the neighboring partition gap (Figure 2B). SRD might also be measured directly through manual or semi-automated segmentation of particular granum elements (membrane, lumen, and partition gap) using high magnifications. Various semi-automated approaches based on pixel gradient and power spectrum analyses were applied by different authors (Kirchhoff et al., 2011; Tsabari et al., 2015; Wood et al., 2018; Li et al., 2020; for details, see Figure 2B). It should be noted that all detailed analyses of granum layers require high-quality images of grana stacks cut parallelly to the vertical granum direction (Figure 2C). The analysis of SRD and sizes of particular granum elements is frequently assessed in studies on the influence of light on the thylakoid ion transport resulting in significant changes in, e.g., lumen or partition gap sizes due to water diffusion and changes in repulsive/attractive forces, respectively (Kim et al., 2005; Puthiyaveetil et al., 2017).

The granum lateral (horizontal) plane analysis requires a precise definition of the granum stack boundary. Due to the high variability of grana architecture in the lateral plane, identifying a single granum stack should be justified each time. For instance, it might be assumed that a single granum stack is characterized by (i) common height, which is shared by all stacked membranes building a particular stack, and (ii) shifted regions which are built by at least three stacked membranes (Figure 2D). In such conditions, membrane overlaps connecting neighboring grana are not considered part of these stacks. Therefore, complicated connections of stacked membranes can be quite easily identified for a reliable analysis (Figure 2D). As a consequence of irregular membrane stacks, the granum diameter established in a single measurement of a layer placed in the middle of the granum should be considered as an oversimplification in most cases. Since a granum stack can be built of layers with significantly different diameters (see examples in Figure 1A), a more precise analysis should be provided, where every layer of the granum stack is measured separately, and the average granum diameter is calculated. Calculations of grana diameters were used to understand the connection between the thylakoid structure and the balance between linear and cyclic electron transport (Wood et al., 2018). Diameter measurements were helpful in the establishment of the structural role of CURT1A proteins (Armbruster et al., 2013) and also, e.g., in deciphering the influence of defective PSII core protein phosphorylation on lateral migration of D1 and FtsH proteins between the membrane domains (Khatoon et al., 2009; Puthiyaveetil et al., 2014).

From the diameter values of all membranes building a single granum, a parameter reflecting irregularity of the particular stack called “Granum Lateral Irregularity” (GLI) can be calculated. GLI is defined as the coefficient of variation (the ratio of the standard deviation to the mean) of membrane diameters within the granum (Kowalewska et al., 2016). The minimal GLI value of 0 is reached by grana stacks built of membranes with the same diameter; the higher variability in granum thylakoid diameters, the higher the GLI value (Figure 2E). GLI as a relative variation gives a good measure of irregularity since the chord error of grana thylakoid diameter is minimalized. GLI parameter, however, does not consider the shifting of membranes in the lateral plane. Therefore, we introduce a new parameter that covers this issue giving information about the irregularity of the granum cross section. “Granum Cross-Sectional Irregularity” (GSI) is calculated by comparing the granum cross-sectional area and rectangle area with the same perimeter and height as the granum cross section (Figure 2F). This approach allows identifying irregular grana whose GLI value is close to 0, while membrane shifting in the lateral plane is significant (Figure 2F). Irregular granum arrangement also results in a substantial increase in the ratio of the granum end-membranes to the total stacked membranes; the more irregular granum, the higher the ratio (Figure 2G). Although the height and diameter of grana stacks significantly increase during the initial stages of chloroplast biogenesis, it was established that granum irregularity decreases during this process, indicating an organized structural pathway of grana maturation (Kowalewska et al., 2016). However, the influence of grana structural irregularity on the thylakoid network structural reorganization in different conditions is entirely unknown and requires further investigation.

## Perspectives

Although the whole thylakoid network of a single chloroplast forms a continuous arrangement, the grana stacks are structurally isolated units whose architecture, resulting from a plethora of interactions between membrane components, might be analyzed quantitatively and qualitatively. Recently, a growing number of studies have shown that the grana nano-morphology itself is a significant factor regulating light harvesting and electron transfer (reviewed in Johnson and Wientjes, 2020). For instance, data derived from quantitative analysis of microscopy images were used to simulate plastocyanin diffusion between stacked and unstacked thylakoid domains. It was shown that a specific range of observed grana diameters results from the optimization of electron transport limited by efficient diffusion of this long-range electron carrier (Hohner et al., 2020). The establishment of a direct role of grana stacks in the efficient performance of photosynthesis has been a subject of many studies, but reasons for the formation of such distinct membrane structures is still under debate (reviewed in Mullineaux, 2005; Anderson et al., 2008; Nevo et al., 2012; Puthiyaveetil et al., 2016; Lambrev and Akhtar, 2019; Moazzami Gudarzi et al., 2021; Müh et al., 2021). An ultrastructure-focused approach adds another dimension to grana function studies, which earlier has been mainly investigated and conceptualized at the level of protein–protein and protein–lipid interactions (reviewed in Johnson and Wientjes, 2020).

In this mini-review, we focused on the use of 2D TEM for quantitative analysis of grana structure. So far, such measurements are mainly executed using manual methods. They are time-consuming and also susceptible to the “human eye” bias, which only partially might be reduced by blinded experiments. The rapid development of machine learning in analyzing different microscopy data (Rawat and Wang, 2017) points to the possibility of applying fully automated protocols for obtaining grana structural parameters. Furthermore, the advantages of the electron microscopy methods *per se* can bring the structural analysis of the thylakoid network to a higher level. Recently developed techniques called jointly “*in situ* liquid cell TEM” could, in the future, enable *in vivo* analysis of the thylakoid network nano-morphology (Pu et al., 2020). On the other hand, a similar goal could be achieved by further developing the 3D structural illumination microscopy method to *in vivo* visualize single layers of grana stacks (Chen et al., 2014). Finally, the advancement in the structural analysis of chloroplast thylakoids should also be extended to the region of STs. Their (i) distinctive role in the light phase of photosynthesis, (ii) complicated and highly organized spatial structure (Bussi et al., 2019), and (iii) possible rearrangements in different genotypes (e.g., Armbruster et al., 2013) point to the importance of detailed structural studies of these thylakoid compartments. However, due to the different architecture of grana and stroma thylakoids, such studies will require the introduction of appropriate structural parameters and new measuring protocols that will consider the complex spatial arrangement of the stroma thylakoids.

## Author Contributions

ŁK and RM provided a conception of the manuscript and prepared figures. ŁK, RM, and AM wrote and edited the manuscript. ŁK provided microscopy images. All authors contributed to the article and approved the submitted version.

## Funding

ŁK acknowledges funding from the National Science Centre, Poland, under grant number 2019/35/D/NZ3/03904.

## Conflict of Interest

The authors declare that the research was conducted in the absence of any commercial or financial relationships that could be construed as a potential conflict of interest.

## Publisher’s Note

All claims expressed in this article are solely those of the authors and do not necessarily represent those of their affiliated organizations, or those of the publisher, the editors and the reviewers. Any product that may be evaluated in this article, or claim that may be made by its manufacturer, is not guaranteed or endorsed by the publisher.
